# Pregnancy rates of day 4 and day 5 embryos after culture in an integrated time-lapse incubator

**DOI:** 10.1186/s12958-017-0253-6

**Published:** 2017-05-08

**Authors:** Verena Holschbach, Julia Weigert, Jens Erik Dietrich, Sabine Roesner, Markus Montag, Thomas Strowitzki, Bettina Toth

**Affiliations:** 1 Department of Gynaecological Endocrinology and Fertility Disorders, Ruprecht- Karls University Heidelberg, Im Neuenheimer Feld 440, 69120 Heidelberg, Germany; 2 ilabcomm GmbH, Eisenachstr. 34, 53757 Sankt Augustin, Germany; 3 Gynecological Endocrinology and Reproductive Medicine, University of Innsbruck, Anichstrasse 35, 6020 Innsbruck, Austria

**Keywords:** IVF, ICSI, Pregnancy rate, Blastocyst culture, Morphokinetic parameter, embryo selection, Time-lapse system

## Abstract

**Background:**

The aim of this study was to compare pregnancy rates in patients undergoing IVF/ICSI with embryo transfer after 4 and 5 days of culture in a closed incubation system with integrated time-lapse imaging.

**Methods:**

Out of *n* = 2207 in vitro fertilization (IVF)/ intracytoplasmic sperm injection (ICSI) cycles performed between January 2011 and April 2016 at a tertiary referral university hospital, a total of *n* = 599 IVF/ICSI cycles with prolonged embryo culture in an integrated time-lapse system (EmbryoScope^©^ (Vitrolife)) until day 4 or 5 were retrospectively analyzed with regard to embryo morphology and pregnancy rates.

**Results:**

A transfer on day 5 compared to a transfer on day 4 did not result in higher implantation and clinical pregnancy rates (IR 29.4% on day 4 versus 33.0% on day 5, *p* = 0.310; CPR 45.2% on day 4 versus 45.7% on day 5, *p* = 1.0). The percentage of ideal embryos transferred on day 4 was comparable to the rate of ideal embryos transferred on day 5 (41.6% versus 44.1%, *p* = 0.508). However, on day 4 a significantly higher number of embryos was transferred (1.92 on day 4 versus 1.84 on day 5, *p* = 0.023), which did not result in higher rates of multiple pregnancies.

**Conclusions:**

Pregnancy rates in IVF/ICSI cycles with integrated time-lapse incubation and transfer on day 4 and 5 are comparable. This finding provides the clinician, IVF laboratory and patient with more flexibility.

**Trial registration:**

This study was retrospectively registered by the local ethics committee of the University of Heidelberg on December 19, 2016 (registration number S-649/2016).

## Background

The majority of embryo transfers (ETs) after in-vitro-fertilization (IVF) is performed at cleavage stages on day 2 or 3 after oocyte retrieval. Activation of the embryonic genome, compaction and blastulation, which are critical developmental steps, have not taken place at that time [1–3]. In animal models, the majority of embryos arrest after transfer to the uterus on day 2 or 3 [2].

Advances in embryo culture conditions, especially the use of sequential media, have allowed a prolongation of embryo culture prior to transfer [4, 5]. A blastocyst culture until day 5, which was first performed in livestock breeding and later in human IVF, offers the advantage of selecting embryos which have completed the crucial steps of compaction and blastulation. Furthermore, an increase in pregnancy rates (PR) was observed after prolonged embryo culture until the blastocyst stage [2, 6–16]. As a consequence, many IVF units established single embryo transfers [11].

Time-lapse systems (TLS or TLI = time-lapse imaging) like the EmbryoScope^©^ may further improve embryo selection while maintaining stable culture conditions [17]. The impact of TLS on pregnancy and miscarriages rates is currently a subject of intensive research.

However, blastocyst culture with transfer on day 5 is not always feasible: embryo transfers sometimes have to be performed on day 4 instead of day 5 due to organizational reasons or suboptimal embryogenesis with imminent developmental arrest or poor embryo quality. In addition, there is evidence of a higher risk for preterm birth [18], large for gestational age (LGA)-babies [19], monozygotic twins [20, 21] and an altered sex ratio [20] as well as concerns about epigenetic alterations and increased congenital anomalies [22, 23] after prolonged embryo culture. Thus shorter ex-vivo-intervals may be favorable in order to minimize neonatal health risks.

Time-lapse imaging might be useful to select embryos before day 5 in order to minimize these potential risks.

Within this retrospective study we compared embryo morphology as well as pregnancy rates following single or multiple embryo transfers on day 4 and day 5 after cultivation in an integrated time-lapse incubator.

## Methods

### Study design

All IVF or ICSI cycles from January 2011 to April 2016 with a prolonged embryo culture until day 4 or 5 at a single university IVF center in Germany were retrospectively analyzed. Only cycles with culture in an integrated time-lapse incubator (EmbryoScope^©^, Vitrolife A/S, Aarhus, Denmark), embryo transfer and documented HCG level were included. Frozen-thaw cycles were excluded.

Patients data included maternal age, causes for infertility, oocyte count, number of fertilized oocytes (pronuclear stages (PN)), day of transfer, stage and quality of transferred embryos, application of IVF laboratory techniques (assisted hatching, calcium ionophore or polar body biopsy), biochemical pregnancy rates, clinical pregnancy rates, presence of fetal heart beat at completed 6 weeks of gestation and the number of frozen PN stages and embryos.

### Study population

The (long and ultralong) agonist as well as the antagonist protocol were applied and oocyte retrieval was performed 36 h after ovulation induction.

Out of *n* = 2207 IVF/ICSI cycles a day 4 or 5 transfer was performed in *n* = 804 cases. Only cycles with culture in an integrated time-lapse incubator (EmbryoScope^©^), embryo transfer, documented HCG level and outcome until 6 completed weeks of gestation were included into the study (*n* = 599 (27.1%)). In 124 IVF/ICSI cycles the embryos were transferred on day 4, 475 transfers were performed on day 5. The decision to transfer the embryo(s) on day 4 instead of day 5 was mainly made for organizational reasons, for example when day 5 was a Sunday or public holiday. The German Embryo Protection Act allows a maximum of 3 embryos to be transferred and limits the number of embryos to be cultured. The transfer day was scheduled depending on the number of pronuclear stage oocytes (PNs) available on day 1. In case the number of available PNs exceeded the number of embryos to be transferred, prolonged culture until day 5 was scheduled with a maximum of 4–5 cultured embryos. Alternatively, day 4 transfer was scheduled in case of organizational reasons (*n* = 90/124 day 4 transfers) or rescheduled to day 4 when only 1–2 embryos were normally developed at day 3 (*n* = 34/124 day 4 transfers).

### IVF laboratory procedures and embryo morphology assessment

Cumulus–oocyte–complexes were isolated and rinsed in Phosphate Buffered Saline (SAGE 4012) supplemented with 0.05% Heparine (Ratiopharm) followed by washing in fertilization medium (Sydney IVF^©^, Cook Medical, Bloomington, IN, USA) and incubation in fertilization medium covered under paraffine oil (Origio, Berlin) in humidified air at 6% CO_2_ and reduced oxygen (5%) in a 4-well dish. Mature metaphase II (MII) oocytes were inseminated by IVF or ICSI. Prior to ICSI oocytes were denuded with hyaluronidase (SynVitro Hyadase^©^, ORIGIO GmbH, Berlin). ICSI was performed at an inverted microscope (Nikon Ti-S^©^, Tokio, Japan) equipped with a heated table and micromanipulators (Mikromanipulator MM89^©^, Narishige, Tokyo, Japan). Spermatozoa were placed into polyvinylpyrrolidone solution (PVP Clinical Grade 10%, Origio, Berlin) in a 60 mm plastic dish (Nunc Microplate^©^, Thermo Fisher Scientific Inc. Waltham, MA, USA). In the same dish, oocytes were placed into drops of gamete medium (Sydney IVF, Cook Medical, Bloomington, IN, USA) and were injected using appropriate micro-capillaries (Micro Injection Pipette^©^ K-MPIP 1035 and Holding Pipette^©^ K-HPIP 1035, Cook Medical, Bloomington, IN, USA). In case of previous fertilization failure or low fertilization rates oocyte activation by calcium-ionophore (Cult-active Ca-Ionophore^©^, Gynemed Medizinprodukte GmbH & Co. KG, Lensahn, Germany) was applied according to the protocol described by Tesarik [24] with modifications according to Montag [25]. Fertilization was assessed 17–19 h after insemination by the presence of two pronuclei (PNs). Culture of embryos was performed in an EmbryoScope^©^ (Vitrolife) at 37 °C, 6.4% CO_2_ and 5.0% O_2_. According to the German Embryo Protection law and dependent on the previous cycles, patients age as well as oocyte quality a maximum of 4–5 embryos were cultured per patient. Supernumerary fertilized oocytes were frozen on day 1 at PN stage.

On day 3 the medium was changed by removing cleavage and adding blastocyst medium (Cook Medical, Bloomington, IN, USA). Assisted hatching procedure was performed in individual cases on day 3 as described by Germond [26] with a 1.48 μm diode laser. Embryo development was described according to the ESHRE Istanbul Consensus Conference [27], Feil [3] for day 4 embryos and blastocyst development was assessed as described by Gardner and Schoolcraft [28].

Two embryos with the best grading and morphokinetic development were selected for transfer. Occasionally, at request of the couple or in cases of certain maternal medical conditions, a single embryo was transferred.

Eight patients received three embryos due to implantation failure in past IVF attempts. Well-developed supernumerary embryos were cryopreserved on the transfer day.

On day 4 an embryo with complete compaction (morula) was defined as “ideal”, on day 5 blastocysts with stage 4 or higher and high grades for the inner cell mass (ICM) and trophectoderm (TE) (AA, AB and BA) quality were considered morphologically “ideal”. Embryos with developmental abnormalities (arrest for >24 h and direct cleavage) were considered as non-ideal albeit matching the above morphologic criteria on the transfer day.

### Pregnancy assessment

Biochemical pregnancies were defined as serum HCG levels ≥10 IU/l on day 14 after oocyte retrieval. Clinical pregnancies were determined by ultrasonographic detection of an intrauterine gestational sac at 5 to 6 weeks of gestation and ongoing pregnancies were defined as presence of a fetal heart beat after completed 6 weeks of gestation. Implantation rates were calculated as described by the ICMART committee 2009 [29].

### Statistics

Data are presented as mean ± standard error of the mean or percentage. Statistical analysis was performed by exact χ^2^–test (Fisher-Yates-Test) and two-sample unpaired t-test for independent samples with SPSS 22.0. *P*-values <0.05 were considered significant.

## Results

### Study population

Patient and cycle characteristics are shown in Table 1. Patient demographics and treatment characteristics including mean maternal age, number of oocytes retrieved, number of injected oocytes in ICSI cycles, number or fertilized oocytes and rate of cycles with special treatments (i.e. calcium ionophore, polar body biopsy or assisted hatching) were not significantly different between the day 4 and day 5 embryo transfer groups.

**Table 1:** Patient characteristics

Patient characteristics	Day 4 transfers	Day 5 transfers	*p*-value
maternal age (years)	35.3 ± 0.37	35.0 ± 0.21	ns (*p* = 0.499)
IVF cycles (n)	31	149	–
ICSI cycles (n)	91	305	–
cycles with splitting IVF/ICSI (n)	2	21	–
oocytes (n)	11.02 ± 0.46	10.91 ± 0.24	ns (*p* = 0.826)
mature oocytes (MII) in ICSI cycles (n)	8.83 ± 0.45	8.67 ± 0.23	ns (*p* = 0.743)
PN/cycle	6.44 ± 0.28	6.16 ± 0.13	ns (*p* = 0.343)
cycles with calcium ionophore	9 (7.3%)	40 (8.4%)	ns (*p* = 0.854)
cycles with assisted hatching	6 (4.8%)	23 (4.8%)	ns (*p* = 1.0)
cycles with polar body biopsy	7 (5.6%)	12 (2.5%)	ns (*p* = 0.087)
no of transferred embryos	SET: N = 12 DET: N = 110 TET: N = 2	SET: N = 82 DET: N = 387 TET: N = 6	s for SET (*p* = 0.038) ns for DET (*p* = 0.061) ns for TET (*p* = 0.673)
mean no of transferred embryos	1.92 ± 0.03	1.84 ± 0.02	s (*p* = 0.023)
transferred ideal embryos	99/238 (41.6%)	385/874 (44.1%)	ns (*p* = 0.508)
transferred embryos with timely developmental stage (day 4 = morula; day 5 = blastocyst)	99/238 (41.6%)	809/874 (92.9%)	s (*p* < 0.001)
frozen PN/cycle	2.04 ± 0.26	1.81 ± 0.12	ns (*p* = 0.388)
frozen embryos/cycle	0.32 ± 0.06	0.37 ± 0.03	ns (*p* = 0.462)

Values are given as mean ± SEM or % (n/total), unless otherwise indicated; *SET* single embryo transfer, *DET* double embryo transfer, *TET* triple embryo transfers, *ns* non-significant, *s* significant (*p* < 0.05)

### Comparison of day 4 and day 5 embryo transfer

**Fig. 1:**
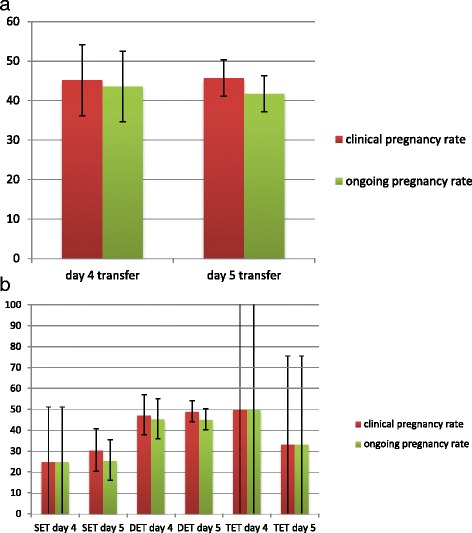
**a** Comparison of clinical and ongoing pregnancy rates of day 4 and day 5 embryo transfers. *Error bars* indicate 95% confidence intervals. **b** Pregnancy rates in correlation to the number of transferred embryos. SET = single embryo transfer; DET = double embryo transfer; TET = triple embryo transfer. *Error bars* indicate 95% confidence interval

There was no significant difference between the biochemical (50.0% for day 4 and 53.1% for day 5), clinical (45.2% for day 4 versus 45.7% for day 5) and ongoing pregnancy rates (43.5% for day 4 vs 41.7% for day 5) between embryos transferred on day 4 or 5 (Table 2, Fig. 1a). Even after exclusion of single and triple embryo transfers, the pregnancy rates of both transfer days were comparable (Table 2, Fig. 1b). Additionally, no significant difference between the implantation rates after day 4 transfers and day 5 transfers could be observed (29.4% versus 33.0% with *p* = 0.310).

**Table 2:** Pregnancy rates by day of embryo transfer

	Day 4 transfers	Day 5 transfers	*p*-value
positive pregnancy test/ embryo transfer	62/124 (50.0%)	252/475 (53.1%)	ns (*p* = 0.547)
positive pregnancy test/ embryo transfer after transfer of 1 embryo	3/12 (25.0%)	30/82 (36.6%)	ns (*p* = 0.531)
positive pregnancy test/ embryo transfer after transfer of 2 embryos	58/110 (52.7%)	220/387 (56.8%)	ns (*p* = 0.448)
positive pregnancy test/ embryo transfer after transfer of 3 embryos	1/2 (50.0%)	2/6 (33.3%)	ns (*p* = 1.0)
clinical pregnancy/ embryo transfer	56/124 (45.2%)	217/475 (45.7%)	ns (*p* = 1.0)
clinical pregnancy/ embryo transfer after transfer of 1 embryo	3/12 (25.0%)	25/82 (30.5%)	ns (*p* = 1.0)
clinical pregnancy/ embryo transfer after transfer of 2 embryos	52/110 (47.3%)	190/387 (49.1%)	ns (*p* = 0.747)
clinical pregnancy/ embryo transfer after transfer of 3 embryos	1/2 (50.0%)	2/6 (33.3%)	ns (*p* = 1.0)
ongoing pregnancy/ embryo transfer	54/124 (43.5%)	198/475 (41.7%)	ns (*p* = 0.759)
ongoing pregnancy/ embryo transfer after transfer of 1 embryo	3/12 (25.0%)	21/82 (25.6%)	ns (*p* = 1.0)
ongoing pregnancy/ embryo transfer after transfer of 2 embryos	50/110 (45.5%)	175/387 (45.2%)	ns (*p* = 1.0)
ongoing pregnancy/ embryo transfer after transfer of 3 embryos	1/2 (50.0%)	2/6 (33.3%)	ns (*p* = 1.0)
multiple pregnancies with > 1 positive heart beat after 6 weeks	11/124 (8.9%) Twins: N = 11 Triplets: N = 0	62/475 (13.1%) Twins: N = 61 Triplets: N = 1	ns (*p* = 0.280)
implantation rate per embryo (no of gestational sacs/no of transferred embryos)	70/238 (29.4%)	288/874 (33.0%)	ns (*p* = 0.310)

*ns* non-significant, *s* significant (with *p* < 0.05)

**Fig. 2:**
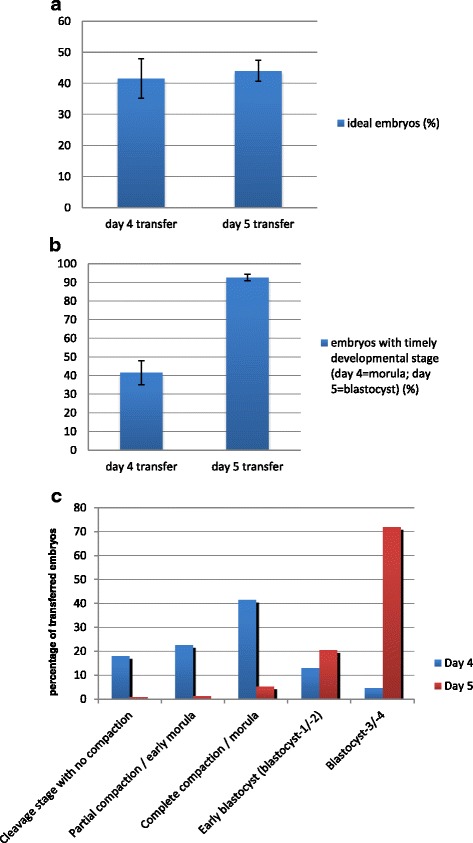
**a** Percentage of ideal (good quality) embryos transferred on day 4 and 5. *Error bars* indicate 95% confidence intervals. **b** Percentage of embryos transferred on day 4 and day 5 with timely development (according to [27]). *Error bars* indicate 95% confidence intervals. **c** Developmental distribution of embryos transferred on day 4 and 5. Day 4 = cycles with embryo transfer on day 4; Day 5 = cycles with embryo transfer on day 5

The rate of ideal embryos transferred did not differ significantly between both groups (41.6% on day 4 versus 44.1% on day 5, *p* = 0.508, Fig. 2a). However, the mean number of embryos transferred on day 4 was higher than on day 5 (1.92 vs 1.84, *p* = 0.023), but the lower number of transferred embryos on day 5 did not lead to significant changes in the rate of ongoing multiple pregnancies (Table 2).

The mean number of cryopreserved embryos was comparable between both groups (Table 1: 0.32 embryos/cycle on day 4 vs. 0.37 on day 5).

## Discussion

A prolonged embryo culture beyond day 3 with transfer of a blastocyst results in a higher implantation rate, possibly due to improved possibilities to judge developmental competence based on enhanced assessment of morphology [30]. It can also give the embryologist time for a preimplantation genetic analysis of polar bodies or trophectoderm if needed [31, 32]. However, an extended embryo culture is not beneficial when embryo selection is not possible [33].

In our study, we compared the outcome of IVF/ICSI cycles with a prolonged embryo culture in an integrated time-lapse culture system and transfer on day 4 to transfer on day 5. In a cohort of 599 transfer cycles, the clinical and ongoing pregnancy rate did not differ significantly between the two groups.

Key disadvantages of a day 4 or 5 culture are a higher cycle cancellation rate, possible epigenetic influences [22] and an increased risk for monozygotic twins [34–36]. The potential negative effect of a longer culture on embryo quality might be caused by an increased exposure of embryos outside the incubator [37], but can be reduced when using closed incubation systems with constant and undisturbed culture conditions (e.g. EmbryoScope^©^) [30].

Usually, a prolonged embryo culture means cultivation until the blastocyst stage on day 5. But organizational reasons (e.g. weekend / public holidays) can lead to the decision to transfer on day 4 instead of day 5.

Current literature evaluating day 4 transfers describes advantages compared to day 2 or 3 transfers [38–41] and no significant difference between day 4 and day 5 transfers with regard to *single* embryo transfers (SET) [3]. The largest prospective study comparing culture durations of 3 to 5 days in a single-step medium showed comparable pregnancy rates in all groups [9] with pregnancy rates of 25.8% after day 4 transfers (*n* = 475) and 27.8% after day 5 transfers (*n* = 694). In a Korean retrospective analysis of 440 IVF cyles with day 4 and 307 cycles with day 5 transfers also no significant difference in pregnancy rates was present [42].

Our study supports the current literature that embryo culture until day 4 seems not to have disadvantages to a 5-days culture, providing more flexibility to clinicians, laboratory staff and patients. Apart from organizational reasons there are other advantages of a 4-days instead of 5-days culture:

1. Transfer on day 4 means that the embryos are placed in the uterine cavity at a time when they are naturally designed to reach the uterus and when uterine contractility is already reduced [43, 44].
2. A shorter time interval in vitro may also reduce the risk of genetic/epigenetic alterations, fetal malformations, monozygotic twinning and preterm birth associated with blastocyst culture [23].

Our study power is slightly limited by the different sample size of the day 4 and 5 study population subgroups (80% of the transfers were day 5 transfers). But this is the first time that pregnancy rates of day 4 and day 5 transfers are compared after cultivation in an integrated time-lapse incubator which ensures very stable culture conditions and can help to identify the most capable embryos by morphokinetic characteristics. In contrast to Lee et al. [42], who made the decision for day 4 transfers in cases of suboptimal culture conditions and therefore transferred a significantly lower percentage of good quality embryos on day 4, we made the decision to transfer on day 4 in the majority of cases already on day 1 in case day 5 was a Sunday or public holiday. Therefore, the bias of suboptimal culture conditions in day 4 transfers was strongly reduced.

Apart from comparable pregnancy rates, we also observed comparable rates of multiple pregnancies after day 4 and day 5 transfers despite a significantly higher number of transferred embryos on day 4 (Table 1, Fig. 1a). Therefore, we calculated the implantation rate per embryo and found that it was slightly lower after a day 4 transfer in comparison to a day 5 transfer (29.4% versus 33.0%, without reaching statistical significance (*p* = 0.310). This finding can be interpreted as evidence that the extended culture of embryos to day 5 enables further de-selection of non-ideal embryos.

## Conclusions

In conclusion, the use of day 4 ETs in non-selected couples results in acceptable pregnancy rates with no difference between day 4 and day 5 transfers in TLS cycles.

Therefore, the decision when to perform the embryo transfer in cycles with a prolonged culture can be based on clinic, patient and IVF laboratory requirements.

## Abbreviations

CPR: Clinical pregnancy rate
ET: Embryo transfer
ICM: Inner cell mass of the embryo
ICSI: Intracytoplasmatic sperm injection
IR: Implantation rate
IVF: In-vitro-fertilization
TE: Trophectoderm
TLI: Time-lapse imaging
TLS: Time-lapse system
